# Hospital Meetings

**Published:** 1904-05-07

**Authors:** 


### HOSPITAL MEETINGS.

### NORTH-WEST LONDON HOSPITAL.

The twenty-fifth annual general Court of Governors and subscribers of this hospital was held on Thursday, 28th ult., the Right Hon. Lord Rathmore, Chairman, presiding.

In submitting the report and statement of accounts for 1903, the Chairman said the work of the hospital had been conducted throughout the year in the usual satisfactory and painstaking way. There were 615 in-patients and 26,079 out-patients, including 6,455 casualty cases. The total number of attendances was 44,465. Twenty-six patients were relieved through the agency of the George Sturge Samaritan Fund, and a large number of out-patients shared in the benefits of the soup-kitchen provided by the treasurer, Mr. George Herring, for the destitute poor of that densely-populated neighbourhood. The problem of finance, the Chairman continued, pressed heavily upon the committee, their position at the close of the year being again one of considerable difficulty. While the income from all sources amounted to £4,539, the expenditure exceeded that sum by £769 15s. 8d., which, added to a deficiency of £1,219 for the previous year, showed a debit balance of nearly £2,000. In view of this, the treasurer addressed an urgent letter to the committee, pointing out the impossibility of continuing the work of the hospital on its present scale without additional outside help, at the same time offering himself to subscribe a sum equal to one-third of the amount raised by any appeal the committee might authorise. The committee very gratefully accepted this munificent offer, and made an appeal to the public which, thanks largely to the generous co-operation of the Press, had produced a fairly satisfactory result, more especially in bringing additions to the list of subscribers. In the absence of the treasurer, he ventured to take the opportunity of publicly expressing the gratitude of the committee—a feeling which was shared by those interested not only in the work of this hospital, but in the work of relieving the sick in the metropolitan hospitals generally, in connection with a similar munificent offer on a larger scale—for the help thus generously given in a time of stress, and to add that none who knew Mr. Herring would doubt his desire that the response to the appeal should be a liberal one. Nor was this the only instance in which remarkable generosity had been shown; the sum of £1,436 19s. 3d. was realised by the Million Pennies Scheme; £750 was received from King Edward's Hospital Fund; £475 12s. 4d. from the Hospital Sunday Fund; £137 11s. from the Hospital Saturday Fund, and a legacy of £500 under the will of the late Mrs. Puseley, while the benefit matinée at the Bedford Palace of Varieties realised £250. This entertainment was under the direct patronage of the Mayor, Aldermen, and Councillors of St. Pancras, and special thanks were due to Mr. B. Pearce Lucas for the use of the hall, and for his munificent support. In conclusion, he desired to thank the ladies (the Misses Learmonth) who carried on the work of this little hospital with so much devotion, as well as the members of the hon. medical staff, and other officers, for their services during the year under review.

The report having been adopted, and other business transacted, Mr. Benjamin Davis called attention to recent police restrictions with regard to collecting-boxes at Friendly Societies' festivals, resulting in a loss of some £30 or £40 to the hospital. After some discussion it was agreed that the secretary should communicate with other hospitals on the subject, and report to the committee. A vote of thanks to the chairman concluded the meeting.

